# “Whatever I said didn’t register with her”: medical fatphobia and interactional and relational disconnect in healthcare encounters

**DOI:** 10.3389/fsoc.2024.1303919

**Published:** 2024-03-22

**Authors:** Carolin Kost, Kimberly Jamie, Elizabeth Mohr

**Affiliations:** ^1^University of Cambridge Centre for Gender Studies, University of Cambridge, Cambridge, United Kingdom; ^2^Department of Sociology, Durham University, Durham, United Kingdom; ^3^Berlin School of Public Health, Charité - Universitätsmedizin Berlin, Berlin, Germany

**Keywords:** fat, fatphobia, medical fatphobia, interaction, cultural health capital

## Abstract

**Introduction:**

This article focuses on medical fatphobia as a specific phenomenon structuring interactions between patients and healthcare practitioners. Throughout the article, we use ‘fat’ and ‘fatphobia’ as the preferred terms in the body positivity and fat acceptance communities. It is well documented that ‘fat’ people frequently experience negative and highly stigmatising healthcare encounters where weight is disproportionately centred and over-attributed as a cause of ill-health. This can compound and worsen disordered eating, trigger mental health problems, and lead to healthcare avoidance. Although the regularity and risks of these weight-focused encounters are well established, there does not yet exist a coherent theoretical framework for understanding such discriminatory practises.

**Methods:**

In this article, we draw on the experiences of 15 fat women who are members of the Health at Every Size (HAES) online community to explore how they perceive their fatness impacting medical encounters.

**Results and discussion:**

Through these data and specifically drawing on the framework of ‘cultural health capital,’ we suggest that given the deep purchase of cultural tropes surrounding it, fatness is perceived to embody and therefore confer on patients’ assumptions of low cultural health capital. We argue that ubiquitously characteristic of medical fatphobia is what we call an ‘interactional and relational disconnect’ between fat patients and healthcare practitioners. We suggest that this disconnect structures fatphobic interactions by over-attributing fatness as the underlying cause of medical problems, which entrenches patient and practitioner ambivalence because of a lack of joint decision-making. We argue that interactional and relational disconnect is produced by, sustained by, and reproduces asymmetric power relations between patients and practitioners. While we demonstrate that patients develop tactics to mitigate and manage fatphobia in healthcare encounters, the persistent interactional asymmetry between doctors and patients means these attempts often fail. We conclude with a plea for sociology to take medical fatphobia seriously as a form of intersectional systemic discrimination. While movements like HAES, fat positivity, and body acceptance create kinship and support fat patients with self-advocacy in healthcare interactions, we suggest that systemic rather than individual change is necessary for effective healthcare inclusion and interaction.

## 1 Introduction

Ellen Maud Bennett died on 11 May 2018, because she was fat. Ms. Benett had been feeling unwell for several years and had repeatedly sought medical intervention. Each time, her symptoms were attributed entirely to her size, she was sent home with advice to return only once she had lost a significant amount of weight. She was offered no support or suggestions about her ill-health beyond weight loss. When the root of her malaise was eventually identified as an inoperable cancer, Ms. Bennett was given just days to live.

While Ms. Bennett’s story became very public—particularly because she used her obituary to call for ‘women of size’ to reject fatness as the primary determinant of their health—it is not a particularly exceptional case (see Kassam, 2018; Ulrey, 2023). Rather, it encapsulates well the persistent and disproportionate focus on weight in healthcare interactions and the potentially devastating implications of such myopism (Brown and Ellis-Ordway, 2021). Indeed, it is well documented by fat studies scholars and activists that fat patients experience poorer interactional exchanges with healthcare practitioners. Fat patients generally have shorter appointment times (Flint et al., 2021), are more likely to be treated discourteously (Aldrick, 2010), and, like Ms. Bennett, are likely to receive generic, banal weight-focused advice rather than robust, tailored medical support (Ananthakumar et al., 2020). Given that fat patients are seen by healthcare practitioners as partially, if not fully, responsible for their own health troubles, they are seen as ‘unworthy of medical time’ and experience delays to specialist referrals and investigative procedures (Ananthakumar et al., 2020, p. 1). Research repeatedly demonstrates that such negative treatment leads to trauma, poorer long-term outcomes (Phelan et al., 2015), internalisation of weight bias (Williams and Annandale, 2019; Davidsen et al., 2023), and in some cases, total avoidance of healthcare encounters (Kost and Jamie, 2022).

Although such endemically poor treatment has been widely documented, there does not yet exist a coherent theoretical framework for understanding such systemic discriminatory healthcare practises. Using the notion of ‘cultural health capital’ (Shim, 2010), we identify the assumptions about fat patients that underpin medical fatphobic practise and describe how it manifests and unfolds in healthcare encounters, sustained by a wider system of healthcare inequality linked with entrenched medical power. As such, we demonstrate that medical fatphobia operates at both expansive and contracted scales, in individual one-on-one healthcare interactions as well as in wider systemic healthcare structures.

## 2 Literature and theoretical context

In this section, we discuss the empirical and theoretical drivers of our article and argument. We provide an overview of empirical research about the extent and nature of fatphobia in healthcare encounters. We then move on to outline the notion of ‘cultural health capital’ (hereafter CHC), which provides a lens through which to understand the disrupting nature of fatness in healthcare interactions. First, we offer some brief reflections on terminology, particularly our use of the term ‘fat’ throughout the article.

### 2.1 A note about terminology

There is a lack of consensus on how to refer to and describe ‘fat’ people in medicine, policy, the media, and society more generally (Bednarek et al., 2023; Jepsen et al., 2023). Descriptors that incorporate terms like ‘overweight’ or ‘obese’ have been widely critiqued by fat scholars and activists because they are based on flawed and simplistic body mass index (BMI) measures. While BMI has some value as a population-level public health tool, it has been widely critiqued for its use to classify individuals as healthy or unhealthy based solely on weight without reference to other factors like exercise or general health (Gutin, 2018). The BMI has also been criticised as rooted in white supremacy and for its disregard of the potential need for different cutoffs for different ethnicities (Maffetone et al., 2017). Indeed, considerable attention has focused on the racist and eugenicist roots of BMI measurements and their contemporary hangovers. In a post for her Your Fat Friend (Gordon, 2019) blog, body positive advocate Aubrey Gordon outlines the ‘bizarre’ history of the BMI, which is wrapped up in mid-century European academic attempts to map and measure ‘the average man’ using research subjects disproportionately recruited from white nations. In this white supremacist model, ‘the average man’ actually means ‘the average *white* man.’ Therefore, Black and minoritised bodies whose composition differs in terms of muscle-to-fat ratio are inaccurately recorded and inappropriately treated when stacked against the white norm of the BMI (Strings, 2019). For Strings (2019), this racist history, coupled with contemporary and historical sexism, feeds into the disproportionate fatphobia faced by people, particularly women of colour. For Redpath (2022), then, BMI ‘must be abolished completely and with immediacy.’ In addition to this over-reliance on BMI measures, terms like ‘overweight’ and ‘obese’ medicalise and problematise particular body types, leading to and compounding anti-fat attitudes. In an effort to temper such overly stigmatising terminology, person-first language has been suggested by various practitioners, groups, and policymakers. Armstrong et al. (2018), for example, argue that using ‘person with obesity’ places the individual before the condition and reduces bias, while Palad and Stanford (2018) argue that such person-first language brings obesity in line with wider medical conventions such as ‘person with diabetes’ or ‘person with autism.’ But such efforts at diplomacy have been wholly rejected by fat individuals, scholars, and activists for continuing to use terminology based on BMI, equating fatness or high weight with ill-health, and locating fatness as a biological dysfunction (Meadows and Daníelsdóttir, 2016).

Avoiding such medicalised and stigmatising language like ‘overweight’ and ‘obese,’ though, risks ushering in well-meaning but largely meaningless euphemisms such as ‘big-boned,’ ‘full-figured,’ or ‘plus-sized’ (Bednarek et al., 2023). Such phrasing others fat people by introducing a degree of implicit comparison to a normative, yet undefined, baseline—‘full-figured,’ for example, implies a deviation from some kind of ‘standard-figured.’ Even if such a standard figure, bone size, or body size exists, it is likely again to be based on simplistic measures like BMI, and it is telling that there are very few polite euphemisms to describe those deviating from it in the other direction who might be described as ‘underweight.’

Despite these debates about phrasing in medicine, media, and policy rumbling on, fat activists are clear in their approach to terminology—just say ‘fat’ (Gordon, 2020), ‘say it loud, say it proud: Fat! Fat!’ (Wann, 1998, p. 18). As a neutral descriptor of body size which avoids socially constructed disease categories of ‘overweight’ or ‘obese,’ using the term ‘fat’ is common practise in the body positivity and fat acceptance communities (Tovar, 2018; Williams and Annandale, 2019). For Meadows and Daníelsdóttir (2016, p. 1), in sanitised and euphemistic phrasing, ‘the apparent need to separate a person from the characteristic in question (i.e., their fatness) implies an inherent adverse judgement.’ Therefore, using ‘fat’ is a way to describe and ‘say my body’s name’ in a way that avoids medicalising and moralising that body (Gordon, 2020). In this way, using the notion ‘fat’ is a political endeavour, enabling fat people to (re)gain control over their own stories and experiences and taking a stance against the negative connotations the term bears in wider society (Saguy and Ward, 2011).

Given this linguistic context, we use the term ‘fat’ throughout the article to describe individuals of higher weights who experience discrimination based on their body size. Moreover, we use the term ‘fatphobia’ as a way to encapsulate the ‘fear, hatred, and loathing of fat bodies’ (Stoll et al., 2022, p. 37), which, like other ‘phobias’ and ‘isms’—sexism, racism, and homophobia—exists at the intersection of and stretches across both individual psychopathologies and cultural and structural perspectives.

### 2.2 Fat patients and healthcare interactions

Despite research demonstrating the medical, social, and cultural complexity of ‘obesity,’ the (mis)conception persists that fatness is a result of poor individual lifestyle choices, apathy, and a lack of willpower (Lupton, 2013). Within this individualised approach, wider structural factors that serve as health determinants become deprioritised, and fatness is located solely as ‘a failure of individual control’ (Brandt and Rozin, 1997, p. 64) and an inability or unwillingness to heed basic public health advice. As a result, fat people are often subject to weight-based stereotyping, stigma, and discrimination in a variety of everyday spaces such as work (Flint et al., 2016), education (Sykes and McPhail, 2008), public transport (Evans et al., 2021), and health-related settings like gyms (Harjunen, 2019), obesity policy (Flint, 2021), and public health campaigns (Department of Health and Social Care Committee, 2022). In short, fatphobia is a broad and deeply embedded societal issue which ‘circulates’ around diverse spaces, rendering them and their social interactions ‘uncomfortable, unwelcoming, unsafe and inaccessible’ (Rinaldi et al., 2020, p. 38). Those who attempt to challenge such fatphobia (e.g., with body positivity) are often met with abuse both online (Kristensen, 2023) and in-person (Johanssen, 2021). Our concern in this article is medical contexts—doctors’ offices, hospitals, nurses’ rooms, and the like. In their narrative review of weight discrimination and its effects, Phelan et al. (2015) demonstrate that fat patients in these settings are subject to discriminatory, stigmatising, and unequal treatment. This biased medical treatment can be seen as partly rooted in the complex and often contradictory story of the medicalisation of ‘obesity.’ On the one hand, Sobal (1995) argues that during the twentieth century, the understanding of fatness as a moral failing decreased in prominence to be replaced by a conception of fatness as a disease. In other words, fatness moved into the medical gaze as a ‘problem’ to be addressed through medical intervention. While such an ontological move might be reasonably expected to garner support or sympathy for fat individuals, the question of agency complicates the picture. Hence, on the other hand, the march of neo-liberal public health models in the latter years of the century placed the focus on individual decision-making and personal responsibility for health. Within this new public health context, the enduring and simplistic ‘calorie intake over expenditure’ model of fatness (Chang and Christakis, 2002) became an issue of individual failure to monitor and control eating habits, contributing to individual ill-health (in the form of ‘obesity’) and a collective ‘obesity crisis.’ Against this backdrop, Lupton notes that practitioners often uncritically adopt wider anti-fat cultural tropes and view fat patients as ‘lazy, stupid, non-compliant and worthless’ (Lupton, 2013, p. 68), embedding and normalising poor treatment of fat patients (Tomiyama et al., 2018). Healthcare practitioners frequently spend less time with overweight patients (Phelan et al., 2015) and are often reported to be rude and disrespectful or even verbally abusive (Ananthakumar et al., 2020). In these shorter consultations, practitioners are also likely to disproportionately focus on weight at the expense of the actual reason for the patients’ visit (Roy et al., 2023) and may ‘weaponize’ weight shame and stigma, albeit ineffectively, to encourage weight loss (Williams and Annandale, 2020).

As well as impacting immediate healthcare interactions, the assumption that health problems result from excess weight also results in practitioners’ reluctance to proceed with investigative procedures (Phelan et al., 2015). A recent review of breast, cervical, and colorectal cancer screening, for instance, demonstrates that practitioners’ unconscious bias against overweight patients places considerable barriers to uptake of screening opportunities (Graham et al., 2022). Aldrick (2010) also reports that one-fifth of obstetrics and gynaecology practitioners express a reluctance to perform pelvic and breast examinations on fat women.

These biases, unsurprisingly, have significant consequences for fat patients. Healthcare interactions are often experienced as stressful, dehumanising, and traumatic (Phelan et al., 2015), which can lead to the internalisation of fatphobia (Davidsen et al., 2023) and, in turn, result in fat patients delaying seeking support or healthcare avoidance altogether. For example, Aramburu and Louis (2002) found that amongst ‘obese’ women, 34% had delayed seeking out medical advice out of fear of being criticised for their weight. Moreover, Ms. Bennett’s story above demonstrates potential iatrogenesis and an increased risk of premature and unnecessary death resulting from fatphobia.

### 2.3 Cultural health capital

Concepts such as ‘weight bias,’ ‘weight discrimination,’ and ‘weight stigma’ have been variously mobilised by scholars and activists to explain this ‘rife’ fatphobia in healthcare practise (Brown and Ellis-Ordway, 2021). These concepts offer useful starting points for understanding the ways that practitioners adopt, internalise, and then play out wider cultural tropes about fatness as a failure of individual control (Nutter et al., 2016). Yet, as Nutter et al. (2016) outline, these notions have been somewhat disparately developed across academic and medical disciplines with divergent priorities, theoretical sensibilities, and end goals. Inasmuch, while these frameworks share some common foci, there does not yet exist a cohesive theoretical core to understand what lies behind endemic discrimination against fat patients and how it is sustained through power relations.

To remedy this, we apply the notion of cultural health capital to understand how healthcare interactions are structured by fatphobia. Following Bourdieu (1986) concept of cultural capital, cultural *health* capital captures ‘how broad social inequalities operate in patient-provider interactions and shape the content and tone of health care encounters’ (Shim, 2010, p. 1). Firmly rooted in the Bourdieusien view that society is deeply hierarchical, Shim (2010) proposes CHC as a particular form of cultural capital that can be leveraged by patients in healthcare interactions to more effectively engage with practitioners. She suggests that particular components of CHC include: medical knowledge based on normative scientific rationale, communication and interaction competency, a proactive and instrumental stance towards health and body, self-discipline, a future-oriented perspective, and an ability to communicate social privilege and resources (Shim, 2010, p. 3). Together, these skills cohere as a ‘toolkit’ for self-presentation, which, depending on providers’ reactions, can positively influence the quality and responsiveness of healthcare interactions, resulting in more attentive care, more equal decision-making, and better outcomes (Dubbin et al., 2013). Like cultural capital more generally, CHC is embedded within social structures and processes of stratification, meaning that patients’ available skills and resources, as well as their ability to acquire and deploy CHC, are impacted by broader inequalities in social structures, institutions, and social life. As such, the acquisition and deployment of CHC is most commonly tacit, accumulated through habitual healthcare practises and experiences that are themselves shaped by wider inequalities of social, cultural, and financial capital. In short, those at the sharp end of health inequalities are likely to possess limited cultural health capital, which constrains the effectiveness of their healthcare interactions and relationships, in turn compounding pre-existing inequality and health determinants.

Broadly speaking, Madden (2015) argues that CHC includes a good knowledge of medical vocabulary, effective communication skills, subscription to neo-liberals of self-discipline, and prioritisation of future health outcomes. A previous study highlights how healthcare practitioners use visual and audio clues to inform their *readings* of patients as dispossessed of these characteristics and, therefore, lacking CHC. Weerasinghe (2012), for example, highlights how immigrant women in Canada experience disrespect, verbal ill-treatment, and exclusion in healthcare interactions based on their audible and visible minority identities. Similarly, the healthcare practitioners in Chang et al. (2015) study describe assuming low CHC in patients with histories of substance misuse. Both examples highlight how macro-level social relations and norms manifest in micro-level healthcare encounters and legitimate power within patient–practitioner interactions (Shim, 2010). Indeed, Shim (2010, p. 4) argues that practitioners ‘do not simply respond to the CHC that patients mobilise’ but are, given their relative power in healthcare encounters, active agents in evaluating and shaping CHC. In this way, patients’ *perceived* CHC is dependent on practitioners’ attitudes towards their individual attributes stemming from wider cultural stereotypes. As such, even when patients *do* have high levels of CHC, practitioners may not recognise or acknowledge this and therefore may fail to give adequate space for patients to deploy their CHC, instead adopting a paternalistic interactional and relational style.

Given the cultural tropes about fat people as lazy, apathetic, and non-compliant with medical advice (Gailey, 2014), we suggest that fatness is often interpreted by practitioners as a visual clue of a lack of CHC. If CHC is rooted in understandings of medical vocabulary, belief in ideals of self-discipline, and investment in the future health outcomes, then deeply engrained anti-fat stereotypes locate fatness as antithetical to possession of CHC (Vartanian et al., 2013; Strings, 2015). As a result, fat patients are understood and treated by practitioners as apathetic, health-illiterate, in need of medical paternalism, and unable to actively participate in their own care decisions (Blackburn and Stathi, 2019). We suggest that this leads to what we call an ‘interactional and relational disconnect’ between fat patients and healthcare practitioners, which is rooted in and reproduces medical fatphobia and is sustained by ‘persistent’ medical power (Pilnick and Dingwall, 2011).

CHC is further compromised in fat *women* due to their deviation from both health and beauty standards (Fikkan and Rothblum, 2012). While fat men comparatively escape or offset aesthetic scrutiny around their body size, women are expected to fulfil the expectation of slimness/thinness, which is perceived as both healthy and attractive (Kwan, 2010). Women who do not comply with this health and beauty ideal are understood as somewhat pathological, failing to fulfil the expectations of self-discipline and adherence to normative biomedical constructions of health and unhealthy bodies. As such, their bodies are positioned as ‘extreme’ (Hockin-Boyers et al., 2020). Fatphobia resulting from the double deviance of being fat *and* a woman permeates healthcare encounters where practitioners meet fat women with bias, dismissiveness, weight focus, and assumptions of fat women having more negative personal qualities (Fikkan and Rothblum, 2012).

Before reporting our findings, we offer an outline of our methodological approach.

## 3 Methods

### 3.1 Study site: HAES groups

This article draws on qualitative data collected in interviews with 15 fat women who were recruited from a private Health at Every Size (HAES) Facebook group. HAES is an ‘alternative public health model’ focused on healthy day-to-day food, exercise, and other practises that aim to achieve good health and wellbeing regardless of weight, body status, or changes (Burgard, 2009, p. 41). Unlike traditional weight-based health models, HAES dispenses with standardised measures—such as weight, BMI, or body fat percentages—which determine categories of ‘healthy’ and ‘unhealthy,’ and instead advocates for a ‘holistic’ approach to health based on intuitive food and exercise practises appropriate to a *personal* sense of health, happiness, and wellbeing (Bacon, 2010). For Robinson (2005), such holism goes hand-in-hand with challenging normative medical ideas of health and weight, whereby HAES (i) embraces natural diversity in body type, shape, and size rather than aiming for a universalised ideal; (ii) acknowledges the long-term ineffectiveness of dieting and weight loss projects; (iii) emphasises the importance of intuitive and relaxed approaches to eating in response to bodily cues rather than external, quantitative targets; and (iv) recognises the contribution of social and emotional wellbeing to overall physical health.

Against this backdrop, HAES online groups are hubs of health-focused information, support, and kinship, offering a space to share advice and experiences of being a fat person, including navigation and negotiation of discriminatory healthcare interactions (Kost and Jamie, 2022). The HAES group from which we recruited our participants is one of the busiest and most diverse in the ‘fatosphere.’ At the time of recruitment, this group had approximately 6,500 members from a range of countries and a variety of socioeconomic, ethnic, and age backgrounds. While participants were sampled from this specific HAES Facebook group, many of them were part of other similar networks, meaning our analysis is not limited to one HAES case study.

### 3.2 Sampling and recruitment

We recruited participants by publishing a post on the group page in October 2019. The post outlined the focus of our research—to understand fat women’s experiences of healthcare interactions and how online groups support them to navigate these encounters—and invited potential participants who met our inclusion criteria to contact CK if they wished to be interviewed. This recruitment post specified that we were seeking women residing in Australia, Canada, the UK, or the USA; aged 25–45 years old; self-defined as middle or upper socioeconomic class; and who described themselves as ‘fat.’

We restricted our recruitment to these countries in contexts where fatness is understood as deviant and where English is the first language. This latter point was important given our focus on participants’ experiences of spoken interactions with healthcare practitioners. We opted for English-speaking countries to ensure alignment between the language of participants’ healthcare encounters and the language of our interviews so that participants could accurately recount stories and avoid the loss of any linguistic nuance. There are, of course, notable differences between the healthcare systems of our participants’ countries which impact interactions and relationships between healthcare practitioners and patients. While Canada and the UK operate universal health coverage models, the USA has a privatised insurance-based system, and Australia adopts a hybrid approach where the government actively encourages private health insurance for higher earners despite universal coverage being in place. As such, care obligations, long-term patient–practitioner relationships, and consumer choice operate differently across these systems. While models of universal coverage traditionally offer less choice to patients about their care (policy shifts towards expanded patient choice notwithstanding), these systems commonly use general practitioners as gatekeepers to provide more specialist services, meaning that patients build sustained relationships over a great many years. Private healthcare systems, in contrast, are often built on fragmented engagements with specialist services accessed directly by patients. In the latter case, where patients are positioned as consumers of services, patients’ dissatisfaction with practitioners can be voiced through withdrawal of custom.

Notwithstanding the differences between these healthcare systems, we recruited participants based on shared experiences of medical fatphobia, which is ubiquitous in Western countries and their medical systems (Lee and Pausé, 2016). We decided on the latter inclusion criteria to control for factors that may compound or complicate participants’ experiences of healthcare interactions, such as racism, classism, or ageism (Crenshaw, 1991 O’Campo and Burke, 2004). Through an analysis of Canadian fiction, for example, Bruusgaard (2021) argues that ageing fat women of middle- and later years are socially positioned as shameful, unfeminine, desexualised and even cautionary tales of unhealthy futures. We take seriously such intersectional prejudices that inevitably come to bear on healthcare encounters and recognise that Black and ethnically minoritised women are disproportionately stigmatised by fatphobia. However, our research to fully understand the basis of medical fatphobia and how it manifests in interactions required a degree of participant homogeneity. We focused only on women because research consistently demonstrates that they are more likely to be stigmatised for being fat (Bordo, 1990) and are more likely to experience medical fatphobia (Anderson et al., 2001).

Upon contacting CK, potential participants received a participant information sheet and a consent form, and a mutually convenient time for the interview was arranged. Our final sample size was 15, comprising two Canadian, 10 US, and 3 British participants. Participants were aged 27–44 years, with a mean age of 35. Although we did not seek an ethnically homogenous sample, all of our participants were white. All participants described themselves as upper- or middle-class and ‘well-educated,’ though we did not specify any particular educational level as a criterion for inclusion.

### 3.3 Data collection and analysis

All interviews were conducted online by CK between October 2019 and January 2020. They lasted 30–60 min and were audio-recorded to be later transcribed verbatim. Interviews were structured by a topic guide, which explored how participants perceived their fatness to impact healthcare interactions and how they used fat positive online communities to navigate these encounters. We have detailed our findings on the latter focus elsewhere, where we argue that online platforms and groups act as spaces of ‘kinship’ based on shared knowledge and experience of medical discrimination (Kost and Jamie, 2022).

We opted for semi-structured interviews to allow participants to freely share their experiences while also enabling us to embed a degree of consistency and comparability between interviews. Given that experiences of medical fatphobia are both deeply personal to a particular individual and relatively consistent in their nature, identifying the ‘data saturation point’ was challenging. While we noticed significant thematic similarities in participants’ accounts after around nine interviews, there was sufficient diversity in participants’ reflections to warrant further data collection. We conducted six further interviews until we were certain we had reached a sufficient saturation threshold, and no new participant inquiries were forthcoming.

Following transcription, the data were analysed thematically using constant case comparison and deviant case analysis. We took an abductive approach to data analysis, whereby we were guided by existing frameworks but also ensured space for novel theoretical understandings to be identified in the data (Tavory and Timmermans, 2014). In particular, we were guided by pre-existing ideas of weight discrimination and bias and more specific conceptualisations of medical fatphobia (Hardy, 2023). At the same time, we retained flexibility to develop and draw in other theoretical ideas to conceptualise the interactional and relational manifestations of such prejudices. While we took a fat positive approach to research, we were careful not to stray into activism in our analysis. We did this by ensuring that our participants’ narratives took centre stage and drove our analysis, keeping all literature (both academic and activist) at arms-length during analysis.

We undertook data analysis in three stages and as both an individual and group endeavour. First, CK conducted open coding to label data with descriptors of its content. This phase allowed participants’ key reflections and experiences to emerge and ensured that CK had a deep familiarity with the data. In the second phase of the analysis, CK organised these descriptive codes into broader themes based on points of confluence in participants’ accounts. To ensure trustworthiness of findings, this phase of analysis utilised constant case comparison, wherein data segments were compared with each other both within individual transcripts and across the whole dataset (Jamie and Pattison Rathbone, 2022). Deviant cases where codes and emerging themes sat in contrast to identified patterns were singled out for specific analysis and to test unfolding findings. The final set of themes and codes emerging from this second stage of research was agreed upon by both CK and KJ in collaboration to ensure robustness. At this stage of analysis, we noticed recurring patterns around assumptions that participants felt practitioners made about their engagement with health and the interactional and relational disruptions that this caused. These patterns in participants’ reflections were reminiscent of other work on cultural health capital, its impacts on healthcare encounters, and the influence of medical power (Shim, 2010). As such, in the third stage of analysis, we proceeded with using this framework to further interrogate the data and more robustly situate our emerging themes within theoretical frameworks for understanding unequal and disrupted healthcare interactions.

### 3.4 Research ethics

The research was given ethical approval from Durham University in September 2019. As per the British Sociological Association (2017), all participants gave fully informed consent prior to beginning their interviews and were informed that they could withdraw without reason. In line with these principles too, during the transcription process, CK fully anonymised participants’ responses by giving participants pseudonyms and removing any details that could be traced back to individuals. Although we closely followed standard sociological procedures for anonymity (i.e., by anonymising the data), there is debate about the anonymisation process in research, which overlaps with activist and political concerns. Allan (2017), for example, describes the desire of her participants in occupied African territories to have their names linked with the personal testimonies they had shared with her. Given that fat studies academic research is often informed by, overlaps with, and is mobilised in fat activism, research such as ours straddles both worlds and, therefore, complicates the question of anonymity. While we adopted a blanket approach to anonymise all our participants’ data, we echo Allan (2017) and others in recognising the need for nuance in approaching the issue of anonymity.

Beyond these universal ethical concerns, interviewing fat participants raised some additional considerations. Given the endemic stigmatisation of fat individuals, traumatic experiences associated with living in a fat body resurfaced several times during interviews, potentially causing psychological distress (Muennig, 2008). We mitigated this risk in several ways. First, the project was carried out within a fat positive framework, which involved not inquiring about participants’ weight and deliberately avoiding any framing of fatness that could seem pathologising. Second, participants were given contact details of mental health support associations in instances where this was deemed appropriate. Third, CK sent a follow-up email to participants after their interviews to ensure they did not suffer negative consequences because of trauma from the interview encounter. This email also served to check participants’ on-going consent. Finally, participants were reminded of CK’s researcher status and encouraged to visit a trained healthcare practitioner for mental or physical health support. Despite the risk of trauma, several participants reported experiencing interviews as something of a therapeutic space for mental healing (see Rossetto, 2014).

## 4 Findings

Below, we report our participants’ experiences of interactional and relational disconnect within healthcare encounters, which is rooted in and stems from fatphobia, the core of which is the assumption that fat patients lack cultural health capital. The relationship between this disconnect, its anti-fat biases, and the role of CHC is circular and interwoven. But we begin our analysis with healthcare practitioners’ assumptions of low CHC as the scaffold structuring fatphobic interactions through sustained medical power.

### 4.1 Embodying low cultural health capital

Participants reported feeling that their fatness was hyper-visible in medical settings and used as a reference point by practitioners to make assumptions about their health behaviours and attitudes (Gailey, 2014). As such, participants felt that their bodies took on a more active and central role in healthcare encounters than would be experienced by other patients. While slim/thin bodies become central to healthcare encounters in particular ways (through their acute dysfunctions) and at particular moments (describing symptoms), participants talked about feeling that their bodies were permanently visible throughout interactions with practitioners. Participants talked about their bodies being visually read as ‘extreme’ (Hockin-Boyers et al., 2020) the moment they entered the medical setting and the rest of the encounter unfolding from this reading. Charlotte, for example, talked about feeling that her body was hyper-visible, while Penelope described her sense that practitioners used her body to make assumptions about her exercise habits and sedentary lifestyle:

*Charlotte: I felt like [my weight] was all anybody saw. […] And so it almost felt like people were just looking at the biggest piece of the puzzle, and that’s all they saw.*

*Penelope: When I walk in, I feel like doctors look at me and immediately make an assumption that this is someone who sits on the couch all day long, [and] does not take care of their health.*

In another example, Dilara talked about significant delays to her eating disorder diagnosis stemming from the hyper-visibility of her fatness and normative assumptions about fat bodies, health, and lifestyles:

*Dilara: My eating disorder went undiagnosed for a really long time. If people had the knowledge of what eating disorders […] look like in children who aren’t thin, I think I would have gotten intervention much earlier. But I did not! And that added years of living with an eating disorder that were really distressing…. Nobody saw the eating disorder because all they saw was that I was overweight.*

Such assumptions about health behaviours were manifested in, carried by, and communicated through participants’ fat bodies, which they felt were dismissed as transgressive, antithetical to good health, and in need of management. This is despite the lived realities in which participants *did* take an active interest in their health and wellbeing. Ilya, for example, described feeling that her exercise routine and interest in health were superseded in healthcare interactions by the visuality of her fatness and the assumptions it produced:

*Ilya: [When entering a doctor’s office], I feel dismissed immediately. There’s a story about my body the minute they meet me. I have had doctors in the past tell me flat-out that they do not believe me that I exercise as much as I do or watch what I eat at all. They […] say you cannot possibly be this size if you work out that much.*

Participants felt that the assumptions made through their bodies rested not solely on surface-level tropes about fat patients’ laziness and apathy but also on suppositions about fat patients’ more fundamental skills and competencies. In other words, participants described feeling that their assumed apathy was not just rooted in assumptions about their *unwillingness* to engage in health but their *inability* to do so owing to a fundamental lack of health-related skills and literacy:

*Charlotte: I said to her, I do not want weight to be part of this consultation. And her response was, I will not bring it up if you do not, just keep your cheat meals to the minimum. I thought that was the most inappropriate and tone-deaf statement, given what I had written and said. I just felt that she did not take me seriously and did not think I was capable of making decisions about my own health.*

*Mary: I have had doctors that kind of ignore my actual question and talk about other things. Usually it’s weight-related—they tell me, your weight can lead to this, this and this. And I’m like, I know, but I’m trying to deal with another acute health issue now! It feels like they think I’ve never heard of the idea that losing weight may be good for my health. It’s ridiculous, really.*

These assumptions about participants’ inability to engage can be understood as an assumption about participants’ lack of CHC, embodied in their fatness. In particular, Shim (2010) and Madden (2015) draw attention to the futurity of CHC, where positive long-term health outcomes are understood, given primacy, and accomplished through adherence to specific routines and ideals. In this way, CHC is not just a set of skills to be mobilised in healthcare encounters for the benefit of better care but, rather, an outlook on health, bodies, and wellbeing that is understood as antithetical to fatness. As such, several participants reported feeling that practitioners had limited faith in their long-term health planning abilities:

*Sarah: I was […] looking for fertility treatment. [Doctor] said, I do not recommend you get pregnant because it is dangerous. […] The whole appointment, he kept hinting at the fact that I had not fully thought this [wish to be pregnant] through and that getting pregnant was an irresponsible, almost reckless decision.*

In being read *from* their bodies, participants felt that low CHC was simultaneously conferred *upon* them too. Given their relative power (Pilnick and Dingwall, 2011), practitioners’ interpretations of participants’ lack of CHC were perceived to become a ‘truth’ at the centre of healthcare encounters. This offered little space for participants to exercise their actual CHC, wherein their autonomous and well-informed choices about their bodies were not given space, and practitioners occupied a paternalistic expert role in unfolding interactions. Mary encapsulated well the relationship between medical power and participants’ CHC:

*Mary: They’re in a position of authority, so you kind of take them up on what they are saying.*

Participants perceived that their compromised CHC created what we call an ‘interactional and relational disconnect’ between themselves and healthcare practitioners, which we explore in the next section.

### 4.2 Interactional and relational disconnect in fatphobic interactions

The interactional and relational disconnect perceived by participants was rooted in a misalignment between participants’ actual health competencies and their desire for egalitarian healthcare interactions where fatness was deprioritised in favour of immediate health concerns, and practitioners’ paternalistic approach, which compromised participants’ CHC and centred fatness as the primary health consideration. This disconnect led to fatphobic interactions, which unfolded in a similar way across participants’ experiences. Participants consistently reported that healthcare encounters disproportionately focused on fatness, which created a sense of ambivalence and rested on and reproduced existing power imbalances.

All participants strongly felt that their fatness was perceived by practitioners as the root cause of many, if not all, of their medical woes. They repeatedly recounted instances when their body size was centred in healthcare interactions, despite them presenting for issues disconnected from their weight. Charlotte demonstrated this well where she described how her struggles with anxiety and depression, which at times lead to suicidal ideations, went undiagnosed for years despite the significant impact they had on her quality of life:

*Charlotte: I felt like [my weight] was all anybody saw. […] Nobody ever asked me about my relationship with food, my body, my depression or anxiety and how that impacted food […]. And so it almost felt like people were just looking at the biggest piece of the puzzle, and that’s all they saw.*

This centring of fatness as the key problem meant that medical encounters were disproportionately focused on weight loss advice. In some instances, participants felt that practitioners positioned weight loss as a kind of panacea, and, as such, they described feeling pressured into reducing their body size, or at least discussing the possibility of doing so:

*Ilya: It seems like he thinks losing weight will magically solve all my issues. This is even though I have repeatedly told him I have no desire to discuss my body size.*

This over-attribution of fatness as a cause of ill-health and the subsequent focus on weight loss advice were perceived to stem from deeply engrained anti-fat biases upheld by policy, media, and medical education. As such, the over-focus on weight in healthcare interactions was understood to be sanctioned even in instances where participants had expressly stated they did not want to discuss weight-related matters. Mary, for example, described an encounter where she had requested not to discuss weight, but the practitioner did so anyway:

*Mary: I straight up told that doctor, I do not want to discuss my weight. I’m in recovery from an eating disorder. And his parting words were that it would really help if I lost weight. It just feels like they cannot help themselves.*

As well as being legitimised through normative tropes of fatness and health, such disregard of participants’ wishes was also perceived to be authorised by fat patients’ compromised CHC and practitioners’ relative power. In other words, the presumption that fat patients lack skills, knowledge, and competencies around health means that practitioners feel more freely able to steer conversations. Lucy summed this up by contrasting her experiences of patient–practitioner communication as a fat patient with her previous experiences before she gained weight:

*Lucy: I gained a lot of weight over the last few years—I have not always been fat. And now, suddenly, I get asked different questions and things are assumed about me that were not […] before. There seems to be a lot less open-mindedness. Gaining weight really has been a turning point in the communication with my healthcare providers.*

Participants reported that keeping the interactional focus on weight left them feeling ambivalent about healthcare interactions. While participants sought help for a specific medical issue and wanted to be treated like ‘thin people,’ they reported feeling disrespected and unheard. This created a tension where participants were often enthusiastic about the principles of patient-centred care and joint decision-making and *wanted* to actively participate in their medical care but were prevented from doing so by practitioners’ directing conversations towards weight. This meant interactions offered limited space for participants to exert their CHC, which in turn compounded their compromised CHC. Indira, for instance, recalled an encounter where the interactional disconnect rooted in fatphobia and the ambivalence it created were particularly notable:

*Indira: I went to my GP because I was experiencing a lot of fatigue and headaches […]. And […] told me that I’d have to go on a diet. And I told her that I’ve tried that before, and if I restrict, I start bingeing. And she just—she did not listen. She just told me that, yeah, well, I can refer you to the local weight loss program. And again, I said, that’s not something I’m interested in. […] And then she said, well, you have to try intermittent fasting and only eat twice a day. […] So it just did not go anywhere. Whatever I said did not register with her.*

In this encounter, despite Indira’s repeated attempts to assert her CHC, steer the conversation to more weight-neutral terrain, and become more actively involved in her care, her practitioner remained disproportionately focused on weight. In some instances, this hyper-focus on weight led to significant long-term healthcare anxiety. In Charlotte’s case, for example, she attributed her current anxiety about healthcare encounters to a lengthy history of medical fatphobia experiences:

*Charlotte: [My doctor] used to be very not compassionate at all. […] She used to say you are going to get diabetes, you are going to have a heart attack because of your weight […] I still have white coat anxiety. Anytime I see a doctor I have elevated blood pressure […]. My GP in Vancouver has learned that they have to put me in the room by myself and do an automatic blood pressure reading because my nervous system is already so heightened just by being in a doctor’s office.*

Others described a sense of frustration that this kind of interaction created, particularly over time as participants became increasingly involved with fat positive online communities and reflected on their history of healthcare interactions as matters of injustice and inequality:

*Ilya: I had surgery to remove this very large cyst from my ovary, and they thought it might be cancer. And my doctor said, all that fat in there is just a skinny woman dying to get out when she was looking at my MRI. And years later, I was, like, are you kidding me?! This is not the time! You should be telling me about the surgery and what to expect.*

While Ilya described her anger about instances of interactional and relational disconnect, other participants described a feeling of resignation and acceptance. Given that attempts to create more equitable interactions often ‘*did not register*’ with practitioners, they commonly failed to create an equitable atmosphere in which fat patients could voice their struggles and find adequate treatment for them. In these instances, like Sarah, participants described reluctantly acquiescing to compromised CHC, exclusion from joint decision-making, and disproportionate focus on fatness:

*Sarah: Over the last year, I’ve kind of just given up. I had this horrible appointment with a gastroenterologist who was very dismissive, violating and not respectful.*

### 4.3 Managing and mitigating fatphobic interactions

Given this context of disrupted fatphobic interactions, participants described developing several tactics to manage and navigate healthcare encounters. For Sarah, managing medical fatphobia meant avoiding healthcare encounters altogether. After a particularly fatphobic previous interaction related to her medications, she described her decision to take matters into her own hands and withdraw from some of her medications despite potential risks:

*Sarah: [My doctor] added a beta blocker to my medication to help with my anxiety […]. That interacts with my antidepressant in a way that I’m, after two minutes of walking, drenched in sweat. It’s really uncomfortable. He of course did not attribute that to the medication though, but to my weight. So I now have, without his advice, withdrawn the medication step by step.*

While Sarah’s decision to forgo medical intervention altogether was an anomaly, all participants reported significantly delaying seeing practitioners. After an earlier ‘*horrible appointment with a gastroenterologist*,’ Sarah reported waiting 6 months until she found the psychological strength to visit another practitioner. Courtney similarly talked about how the constant judgement and hyper-focus on fatness in fatphobic medical encounters left her feeling hesitant to visit practitioners:

*Courtney: I do not even want to engage with [healthcare] because I’m already being scolded from the get-go.*

In their interviews, Carmen and Claudia described having current and worsening health conditions that they were delaying seeking support for because of their anticipation of problematic interactions:

*Carmen: I actually think I am developing arthritis in my right hip, and it’s been going on for a couple of months, and it’s becoming more of a problem. And I know I should go see somebody, but I’m not. I know that one of the first things they’ll tell me is to lose weight. And I do not want to have to have that conversation, so I put off dealing with that.*

*Claudia: If it’s serious enough then […] I think I would raise anything with a doctor. But at the moment I have this issue that I have not seen my doctor for because I know they might blame my size for it. And I know if I was in a smaller body, I probably would not have waited, I would have gone already.*

In both cases, Carmen and Claudia described delaying engaging with healthcare until a problem becomes ‘*serious enough*,’ rather than eschewing medical support altogether. Given that none of our participants had formal medical education, this threshold of ‘*serious enough*’ was somewhat nebulous and idiosyncratic. It was also changeable for different conditions—as Claudia suggested, participants tended to delay seeing practitioners even further for conditions that they thought would be attributed to their weight.

Given their concerns about interactional and relational disconnect, where possible, participants sought out practitioners with whom they were less likely to have fatphobic encounters. For most participants, this process entailed extensively researching local practitioners, collecting recommendations from fat kinship networks (Kost and Jamie, 2022), and trying out a series of different practitioners. Liz explained how initial appointments with potential new practitioners acted as opportunities to assess the likelihood of future medical fatphobia:

*Liz: My first appointment with my primary care was intended to be, kind of, like an interview appointment and not necessarily a full exam because I just wanted to see if I would like to continue seeing her.*

In instances where these initial appointments foreshadowed problematic interactions, participants would move on to try another practitioner. Monica and Lucy described the significant investment of time, effort, emotion, and money that this trial-and-error process involved:

*Lucy: I’d definitely drive out of my way [to find the a non-fatphobic practitioner].*

*Monica: I moved to this area in 2018, and just now [autumn 2019], I have found most of the care team that I needed. And that is with me not working, with me going to appointments at the last minute, whenever, wherever, and unfortunately subjecting myself to abuse to find the right practitioner.*

As such, finding practitioners who were not fatphobic required a level of economic privilege and time commitment that not all participants had. Moreover, given the deep purchase of medical fatphobia and the troublesome interactions it spawns, even participants who had successfully found a supportive healthcare practitioner had to concede to some level of comprised CHC and interactional disconnect. For example, following traumatic experiences with previous practitioners and a lengthy search process, Lorena described finding an obstetrics and gynaecology doctor with whom interactions were only slightly disconnected and who demonstrated some willingness to address medical fatphobia:

*Lorena: I had to find another OBGYN who pushes the idea of weight loss in a subtle way and uses words that I’m okay with and focuses more on behaviours than on weight and will throw out little hints of oh, your weight is down. That’s so good! But at least she is working with me, and she is adjusting her language somewhat to not be an awful healthcare provider.*

These data from Lucy, Monica, and Lorena show that finding non-fatphobic healthcare practitioners is most often unfeasible, both from an individual and systemic perspective. As such, most participants described being left with few options but to visit fatphobic practitioners and to try to develop communication strategies for managing potential interactional and relational disconnect with them. The most common starting point of these strategies was a refusal to discuss weight or weight loss in encounters where these topics were irrelevant:

*Ilya: I have a discussion letting practitioners know that I am not going to discuss weight loss, and that I just want to discuss my medical situation the way they would discuss it with a lower-weight person.*

*Charlotte: If someone talked to me about my weight as a potential contributor to the fact that I have a cold, I’d just be, like, no! And I would just say, I’m not willing to discuss that.*

Participants felt that these kinds of assertions about what they were un/willing to discuss should be enough to ensure effective rather than disconnected interactions. Yet, participants pointed to the need for continual boundary-setting within interactions to ensure that conversations did not take a fatphobic turn. Courtney, for example, talked about the need to be ‘*insistent*’ that practitioners focus on the reason for her visit rather than her fatness:

*Courtney: Sometimes I’ve had doctors dismiss things unless I’m really insistent about it. And so I have to be really insistent that no, this is a problem, and I want you to look at it before you go, and if you do not look at it, I’m gonna make another appointment.*

To keep conversations on a non-fatphobic track and minimise the extent of interactional disconnect, Courtney went on to describe her practise of taking along a pre-prepared ‘script.’ Monica described a similar approach:

*Courtney: And so I also feel like I have in my mind already, if they were [discuss weight] I already know what I would say. And that’s calming.*

*Monica: In some cases I have come up with a list of bullet points. Almost a plan of attack if I am addressed with the weight loss thing, I have to have a plan to counter and advocate for myself.*

Given that participants were aware that practitioners are often not trained to work with fat patients, this preparatory work sometimes went as far as educating themselves on how practitioners could accommodate their bodies during medical procedures:

*Courtney: I had to go to the gynaecologist because I had all these issues going on. And before I went in, I researched what fat people should know at the gynaecologist’s office. I so had things in my mind that I could suggest to my doctor if they were having a problem.*

These attempts by participants to mitigate and manage fatphobic interactions entailed exercising their CHC through demonstrating high levels of health literacy, honed communications, self-advocacy, and endeavours to co-shape healthcare encounters. However, engrained medical fatphobia and the relative power of practitioners to dictate the direction of the conversation (Pilnick and Dingwall, 2011)meant there were limits to the extent that participants could actually challenge and mitigate fatphobia. In particular, the power asymmetry in healthcare encounters meant that participants’ communication and self-advocacy tactics would sometimes crumble in practise. Most commonly, participants described ‘freezing’ when interactions took a fatphobic turn. Although freezing in medical encounters is relatively common for *all* patients, our participants described fatphobia as a particular trigger for their seizing up. In other words, while participants were able to deploy their high CHC to generally navigate and even out healthcare power imbalances, they became powerless when interactions became structured by fatphobia, as Hannah and Mary described:

*Hannah: I just always feel so powerless in medical spaces. Like, I feel like I know what my body needs and is capable of doing pretty well, but when it comes to challenging a doctor’s assumptions or disrespect, I often just feel frozen.*

*Mary: kind of, like, shut down. I just stop talking. It’s like I’m—I’ve gotten good at that first step, this is not weight related, I would prefer not to discuss weight. But once it comes up, I tend to freeze.*

In these instances, despite careful preparation and well-practised scripts, participants freezing in the face of medical power further compromised their CHC by stripping them of their abilities to demonstrate their agency, care, literacy, and investment in health. Lorena demonstrated clearly how an encounter becoming particularly fatphobic led to her freezing and abandoning her communication strategies in a bid to end the encounter as soon as possible:

*Lorena: I was completely shocked. And I immediately almost felt like I was getting beat up on. So I retreated into a defensive posture. And equivocated to get the visit over with as quickly as possible.*

Lorena went on to describe her equivocation as a source of personal frustration, particularly because of the potential for her quietness to be read by the practitioner as acceptance of biomedical models of fatness. Yet, in this instance, Lorena had not given up her shaping the direction of conversation easily. Rather, she had done so to protect her emotional wellbeing. Her story of this encounter demonstrates how tenuous participants’ management and mitigation strategies are in the context of persistent medical power.

## 5 Discussion

Despite repeated appointments with healthcare practitioners over several years, Ellen Maud Bennett died on 11 May 2018, because she was fat. Her obituary drew attention to the ‘fat shaming’ she had experienced in healthcare encounters, which resulted in her tumour going undiagnosed until it became inoperable. In this article, we have used qualitative data from interviews with 15 fat women to illuminate the ways that healthcare encounters involving fat people—particularly fat women—like Ms. Bennett are structured by fatphobia and lead to adverse experiences and outcomes for fat patients. We have argued that ubiquitously characteristic of medical fatphobia is what we call an ‘interactional and relational disconnect’ between fat patients and their practitioners, which over-attributes fatness as the cause of ill-health, leading to ambivalence within health interactions and driving fat patients to potentially risky tactics of management and mitigation.

While this disconnect manifests in one-on-one health encounters, we have demonstrated throughout the article that it is not an issue of individual communication failure. Rather, we have argued that medical fatphobia and interactional and relational disconnect are systemic issues linked with, sustained through, and reproduced by persistent medical power (Pilnick and Dingwall, 2011). In particular, healthcare practitioners’ power enables their readings and assumptions about fat patients’ embodiment of low CHC to become embedded within, and dictate the direction of, medical interactions. We have shown that despite their best efforts at managing and mitigating this embedded fatphobia, fat patients’ tactics of resistance are often stymied by their relative powerlessness.

While scholars have previously drawn attention to the unequal treatment of fat patients in medical encounters, we have mobilised, Shim (2010) notion of cultural health capital as a lens to better locate such treatment as a structural and systemic matter of inequality. We have demonstrated how wider cultural tropes about fat people’s health capital are manifested in individual health interactions and, together with engrained medical power, shape the abilities of fat patients to exercise their health agency, literacy, and engagement in those interactions. By taking this systemic approach and anchoring it in a robust theoretical bedrock, we are moving towards coalescing disparate disciplinary understandings of medical fatphobia. For Nutter et al. (2016), such a coalescence rests on the positioning of medical fatphobia as a social justice issue wherein maltreatment of fat patients can be understood, taken seriously, and addressed in the same way as inadequate healthcare experienced by other marginalised groups.

This social justice approach also requires an intersectional sensibility to illuminate and untangle the ways that fat patients’ other characteristics come to bear on their CHC. In this article, we have concentrated on fat women’s experiences because gender and fatness intersect to produce a double transgression of both health *and* beauty standards, where fat women are read to lack both health and aesthetic capital. But our participants were all able-bodied, white, and described themselves as middle-class, all of which confer a high level of CHC. As such, our argument necessarily misses the intersections between CHC and gender and other characteristics shaping healthcare encounters, such as race. This is not simply a methodological issue but rather one that potentially constructs the ways that interactional and relational disconnect plays out and is managed. In other words, diverse voices in our sample may well have altered our arguments about how bodies are ‘read’ and how these readings are then managed by fat patients. For example, our finding of participants’ refusal to discuss weight and their uses of scripts to shape healthcare encounters may be more complicated for Black women, who are also juggling society’s prejudices and tropes about Black women attempting to engage in self-advocacy. An intersectional approach, coupled with a focus on marginalised groups, would benefit future research by interrogating how different incarnations of systemic discrimination—sexism, racism, ableism, etc.—work together and compound medical fatphobia.

As well as advancing fatphobia and its interactional and relational disconnect as an academic and theoretical interest, closer attention to the role of fatness in health encounters also presents the possibility of a framework for improved patient care. While weight-based ill-treatment of patients has been creeping up the policy and practise agenda in recent years (e.g., Department of Health and Social Care Committee, 2022), calls for better care of fat patients have tended to be couched within a weight loss framework. This approach, in short, rests on the idea that better care that is more attuned to and avoids weight-based discrimination will create environments in which fat patients can be more effectively counselled into weight loss. The focus on more equitable care for fat patients is laudable in these policy and practise drives and clearly echoes our participants’ desires to be fully involved in their care decisions. Yet, the end goal of weight loss in these calls still belies their fatphobic foundations, where fatness is disproportionately constructed as a key medical ‘problem’ to be solved. Moreover, these calls do little to challenge the systemic power asymmetry on which medical fatphobia rests and thrives. A move towards understanding medical fatphobia as a systemic issue connected to health capital and power may present a fruitful scaffold for a more fundamental (re)organisation of these policy drives.

In addition to our fairly homogenous and relatively privileged sample, there are some other limitations to our research. First, we recruited participants from a Health at Every Size social media group, meaning that our participants already had a fairly high level of engagement with issues of medical fatphobia. Moreover, the high number of participants from insurance model healthcare systems wherein patients have more capacity as consumers might have accounted for the particular patterns of resistance and management reported by our participants. Given our ambitions to understand medical fatphobia, how it unfolds, and how fat women find comradeship in online communities (findings reported in Kost and Jamie, 2022), this somewhat partisan sample did not represent too much of a problem. However, future research would benefit from recruiting participants whose views of medical fatphobia are not as shaped by activism or their connections in the fatosphere to obtain ‘naïve’ accounts of fatness in health interactions.

Second, and relatedly, using retrospective interviews always presents the risk that participants misremember or recast particular events. In our case, given participants’ engagement with HAES as a form of fat activism means, it is likely that participants (re)interpreted their health encounters through this specific lens. That is not to say that participants deliberately misrepresented their health encounters or experiences to us during interviews or that their (re)interpretations in collaboration with other HAES members are in any way problematic. However, using retrospective interviews alongside ‘live’ methods like observations or audio-recordings of appointments would enable a more holistic analysis of health encounters where unfolding interactions can be analysed alongside participants’ interpretations of them.

Finally, our analysis is based only on the recollections and narratives of the fat women and makes several inferences about practitioners’ assumptions and motivations without having collected data from practitioners themselves. Given our fat positive stance, we aimed to centre the voices of fat people, which are seldom heard even amongst policy and practise discussions of weight-based discrimination. Notwithstanding this, future research would benefit from gathering data from practitioners to understand medical fatphobia and the role of their own relative power in sustaining it.

## Data availability statement

The raw data supporting the conclusions of this article will be made available by the authors, without undue reservation.

## Ethics statement

The studies involving humans were approved by the Durham University Department of Sociology Ethics Committee. The studies were conducted in accordance with the local legislation and institutional requirements. The participants provided their written informed consent to participate in this study.

## Author contributions

CK: Conceptualization, Data curation, Formal analysis, Methodology, Writing – original draft, Writing – review & editing. KJ: Formal analysis, Supervision, Validation, Writing – original draft, Writing – review & editing. EM: Formal analysis, Writing – original draft, Writing – review & editing.
